# Interferon Potentiates Toll-Like Receptor-Induced Prostaglandin D_2_ Production through Positive Feedback Regulation between Signal Transducer and Activators of Transcription 1 and Reactive Oxygen Species

**DOI:** 10.3389/fimmu.2017.01720

**Published:** 2017-12-04

**Authors:** Ji-Yun Kim, Go-Eun Choi, Hyun Ju Yoo, Hun Sik Kim

**Affiliations:** ^1^Department of Biomedical Sciences, Asan Medical Center, University of Ulsan College of Medicine, Seoul, South Korea; ^2^Institute of Convergence Bio-Health, Dong-A University, Busan, South Korea; ^3^Biomedical Research Center, Department of Convergence Medicine, Asan Institute of Life Sciences, Asan Medical Center, University of Ulsan College of Medicine, Seoul, South Korea; ^4^Department of Microbiology, Asan Medical Center, University of Ulsan College of Medicine, Seoul, South Korea; ^5^Cellular Dysfunction Research Center, Asan Medical Center, University of Ulsan College of Medicine, Seoul, South Korea

**Keywords:** interferon, toll-like receptor, prostaglandin D_2_, signal transducer and activators of transcription 1, reactive oxygen species

## Abstract

Prostaglandin D_2_ (PGD_2_) is a potent lipid mediator that controls inflammation, and its dysregulation has been implicated in diverse inflammatory disorders. Despite significant progress made in understanding the role of PGD_2_ as a key regulator of immune responses, the molecular mechanism underlying PGD_2_ production remains unclear, particularly upon challenge with different and multiple inflammatory stimuli. Interferons (IFNs) potentiate macrophage activation and act in concert with exogenous inflammatory mediators such as toll-like receptor (TLR) ligands to amplify inflammatory responses. A recent study found that IFN-γ enhanced lipopolysaccharide-induced PGD_2_ production, indicating a role of IFNs in PGD_2_ regulation. Here, we demonstrate that TLR-induced PGD_2_ production by macrophages was significantly potentiated by signaling common to IFN-β and IFN-γ in a signal transducer and activators of transcription (STAT)1-dependent mechanism. Such potentiation by IFNs was also observed for PGE_2_ production, despite the differential regulation of PGD synthase and PGE synthase isoforms mediating PGD_2_ and PGE_2_ production under inflammatory conditions. Mechanistic analysis revealed that the generation of intracellular reactive oxygen species (ROS) was remarkably potentiated by IFNs and required for PGD_2_ production, but was nullified by STAT1 deficiency. Conversely, the regulation of STAT1 level and activity by IFNs was largely dependent on ROS levels. Using a model of zymosan-induced peritonitis, the relevance of this finding *in vivo* was supported by marked inhibition of PGD_2_ and ROS produced in peritoneal exudate cells by STAT1 deficiency. Collectively, our findings suggest that IFNs, although not activating on their own, are potent amplifiers of TLR-induced PGD_2_ production *via* positive-feedback regulation between STAT1 and ROS.

## Introduction

Prostaglandins (PGs) are lipid mediators that function as key regulators of immune responses *via* complex interactions with immune cells and parenchymal cells in inflamed tissues (1, 2). They are synthesized through enzymatic conversion of arachidonic acid into PGH_2_ by cyclooxygenases (COXs). While COX-1 is constitutively expressed in most cells, COX-2 expression is inducible and highly upregulated during inflammation, particularly in immune cells (3). Cell-specific PG synthases then catalyze the conversion of PGH_2_ to biologically active PGs including PGD_2_, PGE_2_, PGF_2α_, and PGI_2_ (4). Among them, PGD_2_ and PGE_2_ are major mediators of inflammation and have both pro- and anti-inflammatory effects, depending on the inflammatory stimulus, profile of PGs produced and specific receptor they engage (2, 5–8). PGE_2_ is one of the most physiologically abundant and well-studied PG involved in a wide variety of processes including inflammation and cancer (8). PGD_2_ is a key mediator of inflammatory diseases including asthma (2, 9, 10) and promotes the resolution of inflammation (11–13). PGs are converted from PGH_2_ by different isoforms of PGD synthase (PGDS) and PGE synthase (PGES), respectively (4). Two types of PGDS and three PGES have been identified, referred to as hematopoietic PGDS (H-PGDS), lipocalin PGDS, microsomal PGES (mPGES)-1, mPGES-2, and cytosolic PGES (cPGES), respectively. During an inflammatory response, substantial increases in PGD_2_ and PGE_2_ production are observed and correlate with the infiltration of leukocytes, in which H-PGDS and mPGES-1 play a crucial role (1, 14, 15).

Among the leukocytes, macrophages are a major source of PGs and are vital for the initiation and regulation of inflammation in response to pathogen- or danger-associated molecular patterns (1, 11, 16). Macrophages use pattern recognition receptors such as toll-like receptors (TLRs) to sense these molecular patterns that trigger the production of various inflammatory cytokines, including tumor necrosis factor (TNF)-α and interferons (IFNs) in addition to PGs (17). Recent studies showed that both PGD_2_ and PGE_2_ are produced by macrophages upon exposure to lipopolysaccharide (LPS), a TLR4 ligand (18), with a differential requirement for reactive oxygen species (ROS) (14). These results suggest that macrophages exposed to various stimuli are a suitable cellular model for studying the mechanism underlying PGD_2_ and/or PGE_2_ production under inflammatory conditions. As inflammatory mediators, ROS have emerged as major regulators of inflammation processes and multiple signaling pathways including the PG synthesis pathway (19). A previous study showed that NADPH oxidase (NOX)-derived ROS regulates COX-2 expression and subsequent production of PGE_2_ in LPS-activated microglia (20). However, a recent study reported that PGD_2_, but not PGE_2_, production depends on NOX2-derived ROS in LPS-stimulated bone marrow-derived macrophages (BMDMs) (14). Thus, the involvement of ROS in PGD_2_ and PGE_2_ synthesis remains unclear.

Interferons are key cytokines that control macrophage activation, inflammation, and host defense (21–23). They are subdivided into type I (IFN-α/β), type II (IFN-γ), and type III (IFN-λ) IFNs. They principally signal through the Janus kinase (JAK)/signal transducer and activators of transcription (STAT) pathway and require STAT1 in common for target gene transcription (24–26). The significance of STAT1 in IFN-α/β and IFN-γ signaling was established in studies using STAT1-null mice (27, 28) and mutant cell lines (25). Studies of germline human STAT1 mutation also revealed abrogation of STAT1-dependent responses to both IFN-γ and IFN-α (29, 30). A recent study demonstrated that LPS-mediated activation of STAT1 and STAT3 contributes to COX-2 expression and PGE_2_ production in microglial cells (31). STAT1 was also required for PGE_2_ production by follicular dendritic cell (DC)-like cells exposed to IFN-γ (32). While IFN-γ enhanced the production of PGD_2_ in LPS-stimulated DCs (33), the involvement of STAT1 in this context has not been characterized. In addition, the regulation of PGD_2_ production by type I IFNs and STAT1 pathway has not yet been investigated. Furthermore, several studies revealed that STAT1 not only regulates ROS generation (34, 35) but also is activated by ROS through phosphorylation (36, 37), suggesting that functional cross-talk occurs between STAT1 and ROS. To better understand the role of IFN/STAT1 pathway in PGD_2_ production, we explored the outcomes and underlying mechanism of PGD_2_ production in comparison with PGE_2_ production in the context of combined IFN and TLR stimulation in STAT1-null macrophages. Our results revealed that IFNs (IFN-β, IFN-γ) alone are not triggers but are potent amplifiers of PGD_2_ production in TLR-stimulated macrophages *via* positive-feedback regulation between STAT1 and ROS, thus positioning IFNs as key regulators of PGD_2_ production under inflammatory conditions.

## Materials and Methods

### Mice

C57BL/6 mice were purchased from Jackson Laboratory (Bar Harbor, ME, USA). STAT1-null mice were from Taconic Farms and were backcrossed to C57BL/6 mice for 12 generations (34). Eight- to 10-week old C57BL/6 wild-type (STAT1+/+) and age-matched STAT1-null (STAT1−/−) mice were used in this study. All mice were maintained under specific pathogen-free condition.

### Ethics Statement

All animal experiments in this study were approved by and conducted in accordance with the approved guidelines of the Institutional Animal Care and Use Committee (IACUC) of Asan Institute for Life Science with license number of 2012-02-074.

### Macrophage Preparation and Culture

Peritoneal macrophages were isolated from 8- to 10-week-old mice by peritoneal lavage, 3 days after intraperitoneal (i.p.) injection of 3.85% thioglycolate (Difco, Detroit, MI, USA), plated on plastic tissue culture plates, and incubated at 37°C for 3 h. Non-adherent cells were removed by three washes with fresh Dulbecco’s modified Eagle’s medium, and the adherent macrophages were cultured overnight in Dulbecco’s modified Eagle’s medium supplemented with 10% fetal bovine serum, 100 U/mL penicillin, 100 µg/mL streptomycin, and 2 mM glutamine.

### Reagents and Antibodies

Pam3CSK4 (TLR1/2) and LPS (TLR4) were obtained from InvivoGen (Carlsbad, CA, USA). Mouse recombinant IFN-β was purchased from PBL InterferonSource (now PBL Assay Science, Piscataway, NJ, USA) and mouse IFN-γ was from R&D Systems (Minneapolis, MN, USA). Antibodies used in this study were obtained as follows. Anti-H-PGDS and Anti-COX-2 antibodies were produced by Cayman Chemical (Ann Arbor, MI, USA). Anti-STAT1 and anti-pY701-STAT1 antibodies were from Cell Signaling Technology (Danvers, MA, USA). Anti-actin antibody was from BD Biosciences (Franklin Lakes, NJ, USA). PGs were purchased from Cayman Chemical (Ann Arbor, MI, USA). All other chemicals were from Sigma (St. Louis, MO, USA), unless stated otherwise.

### PG Measurement by LC-MS-MS

A LC-MS/MS system equipped with a 1290 HPLC (Agilent, Santa Clara, CA, USA), Qtrap 5500 (ABSciex, Framingham, MA, USA), and reverse phase column (Pursuit 5 200 mm × 2.0 mm; Agilent) was used. The separation gradient for PGD_2_ and PGE_2_ analysis used mobile phase A (0.1% acetic acid in H_2_O) and mobile phase B [0.1% acetic acid in acetonitrile/methanol (84/16, v/v)] and proceeded at 300 µL/min and 25°C. The separation gradient was as follows: 40–60% of B for 5 min, 60–100% of B for 0.1 min, hold at 100% of B for 4.9 min, 100–40% of B for 0.1 min, and then hold at 40% of A for 2.9 min. Multiple reaction monitoring mode was used in negative ion mode, and the extracted ion chromatogram corresponding to the specific transition of each analyte was used for quantification (Q1/Q3 = 351/271 and 351/315, retention time (RT) = 4.34 min for PGE_2_; Q1/Q3 = 355/275 and 355/319, RT = 4.34 min for PGE_2_-d4; Q1/Q3 = 351/271 and 351/315, RT = 4.66 min for PGD_2_; Q1/Q3 = 355/275 and 355/319, RT = 4.66 min for PGD_2_-d4). The calibration range for each analyte was 0.1–1000 nM (*r*^2^ ≥ 0.99).

### RNA Analysis

Total RNAs from macrophages that had been left or stimulated with Pam3CSK4 with or without IFN (IFN-β or IFN-γ) for the indicated times were isolated using TRIzol reagent (Invitrogen, Carlsbad, CA, USA). cDNA was synthesized from 1 µg RNA using the ReverTra Ace qPCR RT kit (Toyobo, Osaka, Japan) according to the manufacturer’s instructions. RNA analysis using real-time PCR was performed as described previously (38). The following PCR primers were used: 5′-TGG GAA GAC AGC GTT GGA G-3′ (forward) and 5′-AGG CGA GGT GCT TGA TGT G-3′(reverse) for mouse H-PGDS; 5′-CTG CTG GTC ATC AAG ATG TAC G-3′ (forward) and 5′-CCC AGG TAG GCC ACG TGT GT-3′ (reverse) for mouse mPGES-1; 5′-AAG ACA TGT CCC TTC TGC-3′ (forward) and 5′-CCA AGA TGG GCA CTT TCC-3′ (reverse) for mouse mPGES-2; 5′-AGT CAT GGC CTA GGT TAA C-3′ (forward) and 5′-TGT GAA TCA TCA TCT GCT CC-3′ (reverse) for mouse cPGES; 5′-CTT TGC ACA ACA CTT CAC CCA CC-3′ (forward) and 5′-AGC AAC CCA AAC ACC TCC TGG-3′ (reverse) for mouse COX-1; 5′-GCA TTC TTT GCC CAG CAC TT-3′ (forward) and 5′-AGA CCA GGC ACC GAC CAA AGA-3′ (reverse) for mouse COX-2; 5′-AGT ATG ATG ACA TCA AGA AGG-3′ (forward) and 5′-ATG GTA TTC AAG AGAGTA GGG-3′ (reverse) for mouse GAPDH.

### Western Blot Analysis

Macrophages were lysed in lysis buffer [50 mM Tris–HCl (pH 7.5), 150 mM NaCl, 1% Triton X-100, 5 mM EDTA, 0.5 mM Na_3_VO_4_, 50 mM NaF, 1 mM PMSF, and protease inhibitor cocktail (Thermo Fisher Scientific, Waltham, MA, USA)] for 30 min on ice. Cell debris was removed by centrifugation at 20,000 × *g* for 15 min at 4°C. Protein concentration in cell lysates was determined using a Bio-Rad protein assay kit (Hercules, CA, USA). An equal amount of protein for each sample was separated by 8 or 10% SDS-PAGE and subsequently transferred onto PVDF membrane (Millipore, Billerica, MA, USA). After blocking with 5% skim milk for 1 h in TBS-T (0.1% Tween 20 in TBS), membranes were incubated with primary antibodies and then with the respective HRP-conjugated secondary antibody (Santa Cruz Biotechnologies, Santa Cruz, CA, USA). Blots were developed with SuperSignal West Pico (Pierce, Rockford, IL, USA) and signals were detected with LAS-4000 (Fujifilm, Tokyo, Japan).

### Measurement of ROS

Intracellular ROS levels were determined as previously described with some modifications (34). To measure superoxide anions and peroxide, macrophages were loaded with 5 µM chloromethyl derivative of 2′,7′-dichloro-dihydrofluorescein diacetate (CM2-DCFDA, Molecular Probes, Eugene, OR, USA) and 20 µM dihydroethidium (DHE, Molecular Probes), respectively, in HBSS for 30 min at 37°C.

#### Confocal Analysis

Confocal analysis was performed with cells on coverslips (Marienfeld-Superior, Lauda-Königshofen, Germany) placed in 6-well plates (Nunc, Roskilde, Denmark). After staining with CM2-DCFDA or DHE, macrophages were fixed with 10% methanol for 5 min. Cells were imaged using a LSM 710 laser-scanning confocal microscope (Carl Zeiss AG, Oberkochen, Germany).

#### Flow Cytometry

After each treatment, cells were washed with cold PBS and resuspended in 0.5 mL PBS supplemented with 1% fetal bovine serum. Accumulation of intracellular fluorescence was analyzed by using Accuri C6 (BD Biosciences).

### Zymosan-Induced Peritonitis Model

Acute peritoneal inflammation was induced as described previously with slight modifications (13). Zymosan A (Sigma-Aldrich) was freshly prepared (2 mg/mL) in sterile 0.9% w/v saline and 0.5 mL was injected intraperitoneally (i.p.). Animals were sacrificed at the indicated time points. The peritoneal cavity was lavaged with 1 mL of saline, and after 30 s of gentle manual massage the exudate was retrieved and centrifuged at 400 × *g* for 10 min. Cells were counted with a hemocytometer and the supernatants were frozen at −70°C prior to further analysis.

### Statistical Analysis

All data were analyzed using GraphPad Prism v.4.00 (GraphPad Software, Inc., La Jolla, CA, USA). The results are presented as means ± SD. Student’s *t*-test (unpaired, two-tailed) was employed, and statistical significance was defined as *P* < 0.05.

## Results

### STAT1 Regulates PGD_2_ and PGE_2_ Production

Interferons are well-known for their ability to potentiate macrophage activation for cytokine production through the STAT1-dependent pathway. To explore the role of STAT1 in PG production under inflammatory conditions, we first assessed the production of PGD_2_ in comparison with that of PGE_2_ by TLR-stimulated peritoneal macrophages. A recent study reported that both PGD_2_ and PGE_2_ are produced by macrophages in response to TLR4 ligand LPS (14, 15, 18). In addition to the production of inflammatory cytokines in a MyD88-dependent pathway, TLR4 engagement by LPS can induce the production of IFN-β in a TRIF-dependent manner (39). For comparison, we also used Pam3CSK4, a TLR1/2 ligand, as a stimulus that is linked to the production of cytokines, but not of IFN-β (40–42). After stimulation with Pam3CSK4, comparable levels of PGD_2_ and PGE_2_ were produced by wild-type and STAT1-null macrophages (Figure S1 in Supplementary Material). Notably, significant differences were observed for both PGD_2_ and PGE_2_ production between wild-type and STAT1-null macrophages exposed to LPS (Figure S1 in Supplementary Material). LPS treatment substantially augmented PG production by wild-type macrophages, which was not the case for STAT1-null macrophages, suggesting the involvement of the IFN-β/STAT1 pathway in this context.

**Figure 1:**
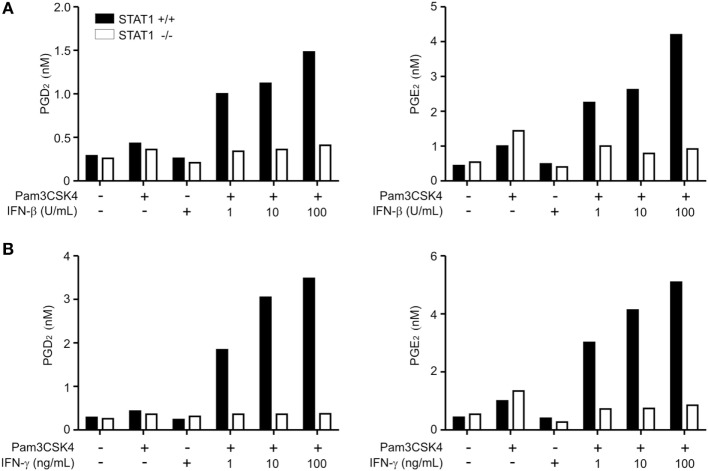
Production of PGD_2_ and PGE_2_ is regulated by signal transducer and activators of transcription 1 (STAT1). Peritoneal macrophages from wild-type or STAT1-knockout mice were left untreated (−) or treated with Pam3CSK4 (1 µg/mL) for 24 h with or without the indicated concentrations of interferon (IFN)-β **(A)** or IFN-γ **(B)**. The production of PGD_2_ and PGE_2_ in the culture supernatant was determined by ELISA. The results shown are from a representative experiment of three biological replicates.

Next, to determine the direct contribution of IFN-β to PG production, we employed Pam3CSK4 as a standard stimulus to assess the macrophage response to IFN-β. IFN-β substantially and dose-dependently augmented both PGD_2_ and PGE_2_ production from Pam3CSK4-treated wild-type macrophages, whereas it had no effects on STAT1-null macrophages (Figure 1A). IFN-β alone did not trigger PG production over control levels, suggesting a potentiating, rather than triggering, role of IFN-β in PG production. STAT1 is the principal signaling mediator of IFN-γ, originally termed as macrophage-activating factor, as well as IFN-β (21, 43). Accordingly, we evaluated whether the dependence of PG production on STAT1 occurred in response to IFN-γ co-treatment. We found similar substantial and dose-dependent enhancement of PG production upon IFN-γ co-treatment in a STAT1-dependent manner (Figure 1B). Thus, these results suggest that the STAT1-dependent pathway common to IFN-β and IFN-γ stimulation potently augments TLR-induced PGD_2_ and PGE_2_ production by macrophages.

### Differential Regulation of H-PGDS and mPGES-1 by STAT1

**Figure 2:**
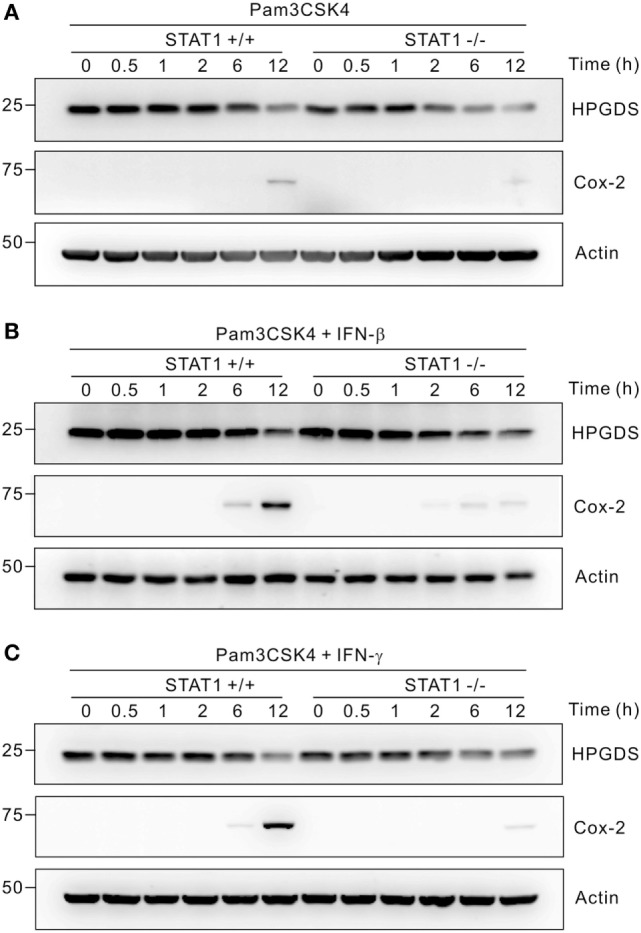
Regulation of hematopoietic PGDS (H-PGDS) is STAT1-independent. Peritoneal macrophages were incubated with 1 µg/mL of Pam3CSK4 for the indicated times in the absence **(A)** or presence of 100 U/mL of interferons (IFN)-β **(B)** or 100 ng/mL of IFN-γ **(C)**. Cells were then lysed, and the lysates were immunoblotted for H-PGDS, cyclooxygenase (COX)-2, and actin. Actin was used as a loading control. A representative result of three biological replicates is shown.

To gain insight into the underlying mechanism, we investigated whether STAT1 affected the expression of enzymes involved in PGD_2_ and PGE_2_ biosynthesis. PGD_2_ and PGE_2_ are synthesized by stepwise involvement of COXs and then various isoforms of PGDS and PGES, respectively. In LPS-stimulated macrophages, H-PGDS and mPGES-1 were shown to be major sources of PGD_2_ and PGE_2_ production, respectively (14, 15). As shown in Figure S2 in Supplementary Material, we observed that both IFN-β and IFN-γ mediate time-dependent increases in the mRNA levels of mPGES-1 and COX-2 in Pam3CSK4-treated wild-type macrophages; this augmentation was abrogated by STAT1 deficiency. These results were compatible with previous findings showing highly inducible expression of mPGES-1 and COX-2 following treatment with inflammatory stimuli (44, 45). In comparison, the mRNA levels of mPGES-2, cPGES, and COX-1 were not affected by IFN treatment and STAT1 deficiency. Notably, unlike the mRNA levels of mPGES-1, IFN-β and IFN-γ did not affect the mRNA expression of H-PGDS at all time points tested in Pam3CSK4-treated macrophages regardless of STAT1 deficiency. Consistent with these results, the protein levels of H-PGDS were nearly unaffected by treatment with Pam3CSK4 and/or IFNs and were comparable between wild-type and STAT1-null macrophages (Figure 2). In contrast, the protein expression of COX-2 was highly inducible by co-treatment with IFNs in a STAT1-dependent manner. These results suggest that STAT1 regulates PG production differentially at the level of PG synthases (i.e., H-PGDS and mPGES-1), despite its critical requirement by IFNs for PGD_2_ and PGE_2_ production.

### STAT1 Regulates ROS Generation

**Figure 3:**
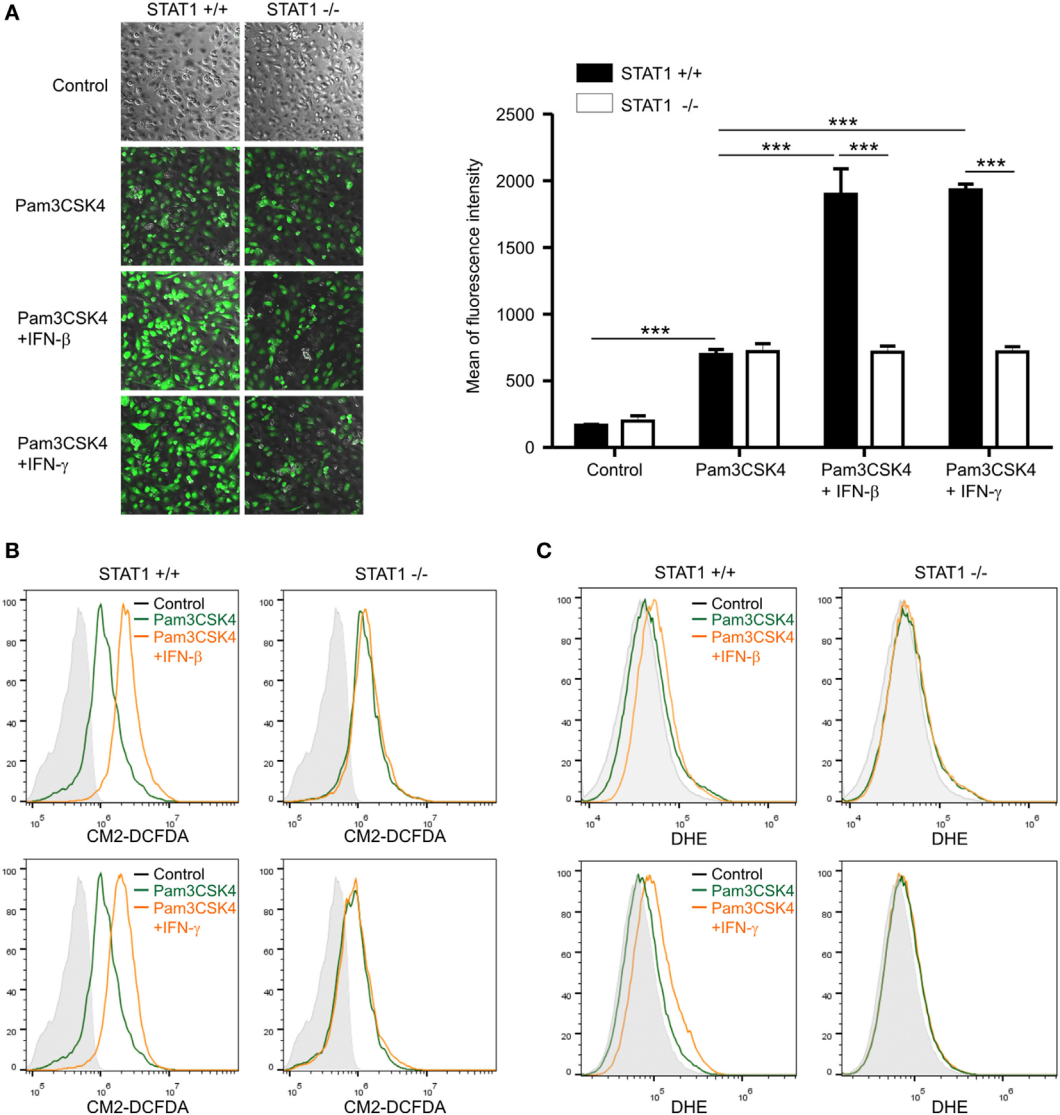
Reactive oxygen species (ROS) generation is regulated by signal transducer and activators of transcription 1. Peritoneal macrophages were left untreated (control) or incubated with 1 µg/mL of Pam3CSK4 in the absence or presence of interferon (IFN)-β (100 U/mL) or IFN-γ (100 ng/mL) for 6 h. Macrophages were then stained for 30 min with 5 µM of CM2-DCFDA **(A,B)** or 20 µM of DHE **(C)**. **(A)** After staining, cells were visualized with a phase-contrast microscope or confocal fluorescence microscope and fluorescence intensity was measured. Data represent the means ± SD from quadruplicate samples. ***P < 0.001. **(B,C)** ROS generation was analyzed by flow cytometry from duplicate samples in each group. Shown are the representative results of at least three independent experiments.

To study the mechanism of STAT1-dependent PGD_2_ production by IFNs, we next assessed the effect of STAT1 on ROS generation. ROS have been implicated in the induction of COX-2 expression and PG production (14, 19, 20), and ROS generation could be regulated by STAT1 in a NOX-dependent manner (34, 35). First, we investigated the levels of ROS in wild-type and STAT1-null macrophages upon treatment with Pam3CSK4 and/or IFNs. To detect ROS, we used the hydrogen peroxide/hydroxyl radical-specific fluorescent probe CM2-DCFDA. By using confocal microscopy, we observed that Pam3CSK4 alone induced moderate but similar increases in intracellular ROS levels in wild-type and STAT1-null macrophages (Figure 3A). In comparison, co-treatment with IFN-β or IFN-γ caused further and significant increases in ROS generation in wild-type macrophages, but not in STAT1-null macrophages (Figure 3A). For more quantitative measurement of ROS generation, flow cytometry analysis was then performed. Similar to the results observed by confocal microscopy, ROS generation was moderately induced by Pam3CSK4 alone and profoundly increased by co-treatment with IFN-β or IFN-γ in wild-type macrophages, which was not the case for STAT1-null macrophages (Figure 3B). To confirm these findings, we also used the superoxide anion-specific fluorescent probe DHE, which is commonly used to analyze respiratory bursts in phagocytes. Similar and STAT1-dependent regulation of superoxide levels were observed in macrophages exposed to Pam3CSK4 and/or IFNs (Figure 3C). Consistent with the potentiating role of IFNs, IFN-β or IFN-γ alone did not trigger ROS generation in macrophages, as determined by CM2-DCFDA and DHE staining (Figure S3 in Supplementary Material). Taken together, our results suggest the regulation of ROS generation by STAT1 as a potential mechanism underlying IFN-dependent PGD_2_ production in macrophages.

### ROS Regulate the Production of PGD_2_ and PGE_2_

**Figure 4:**
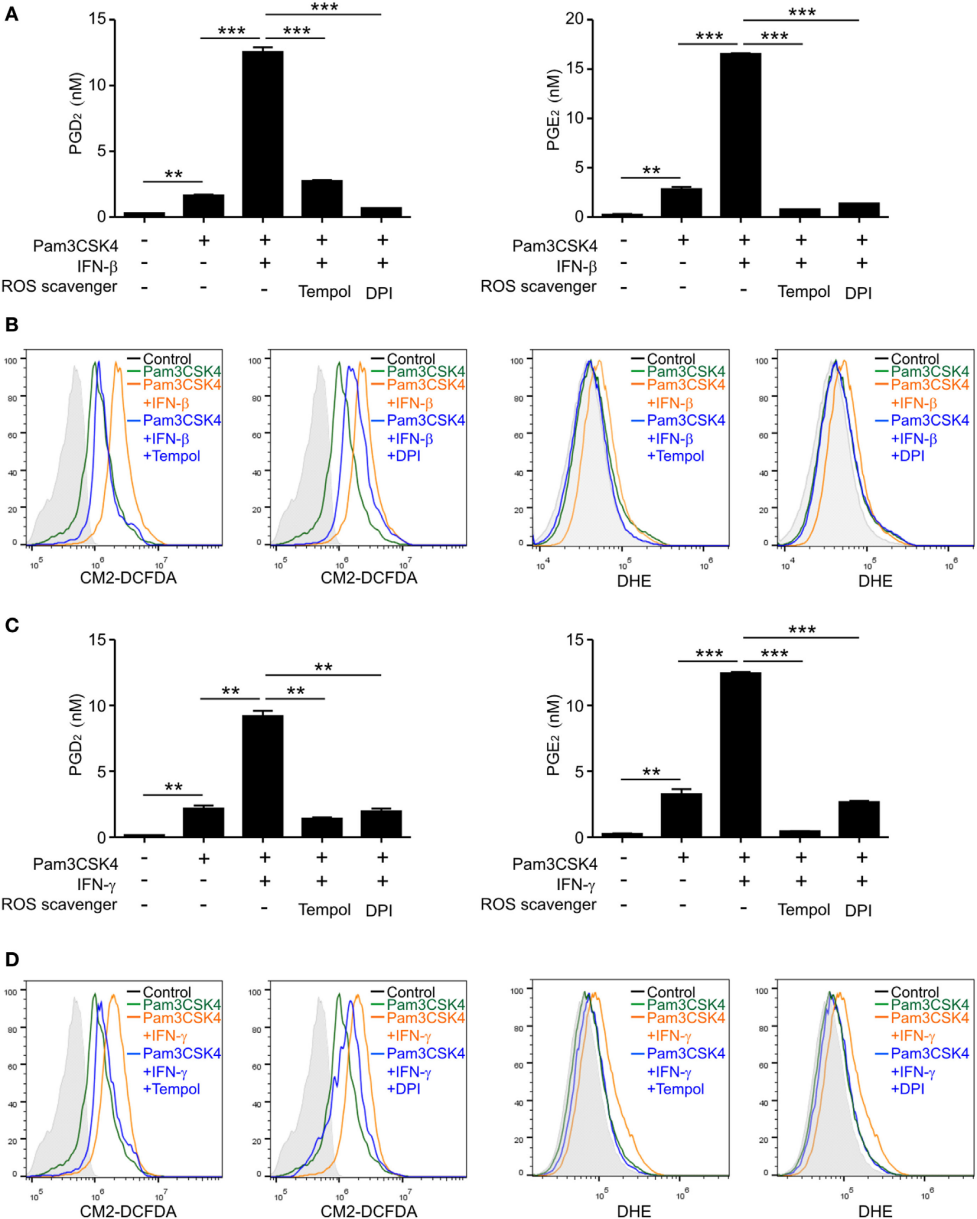
Reactive oxygen species (ROS) generation is required for PGD_2_ and PGE_2_ production. Macrophages were pretreated for 30 min with or without ROS scavengers, Tempol (10 mM), or diphenylene iodonium (DPI) (5 µM), and then left or stimulated with Pam3CSK4 (1 µg/mL) in the absence or presence of Interferon (IFN)-β (100 U/mL) **(A,B)** or IFN-γ (100 ng/mL) **(C,D)**. **(A,C)** After 24 h of stimulation, the concentrations of PGD_2_ and PGE_2_ in the culture medium were determined by ELISA. Data represent the means ± SD from duplicate samples. **P < 0.01; ***P < 0.001. **(B,D)** After 6 h of Pam3CSK4 treatment with or without 100 U/mL of IFN-β **(B)** or 100 ng/mL of IFN-γ **(D)**, macrophages were stained with CM2-DCFDA or DHE and fluorescence intensity was analyzed by flow cytometry from duplicate samples in each group. Shown are the representative results of at least three independent experiments.

Having observed STAT1-dependent ROS generation by IFNs, we next investigated whether ROS in this context are required for PGD_2_ and/or PGE_2_ production. To this end, we used Tempol, a stable and cell-permeable superoxide dismutase mimetic that acts as a free radical scavenger. Given an implication of NOX in the regulation of PG production (20), we also used diphenylene iodonium (DPI), a commonly used NOX inhibitor, to scavenge intracellular ROS. Pretreatment with Tempol or DPI abolished the production of PGD_2_ and PGE_2_ by macrophages treated with the IFN-β/Pam3CSK4 combination (Figure 4A), which correlated with intracellular ROS levels (Figure 4B). Similarly, the production of PGD_2_ and PGE_2_, along with ROS generation, by combined stimulation with IFN-γ and Pam3CSK4 was greatly diminished by Tempol or DPI pretreatment (Figures 4C,D). These results suggest that ROS are required and common mediators of IFN-mediated production of PGD_2_ and PGE_2_.

### ROS Regulate STAT1 Activation

**Figure 5:**
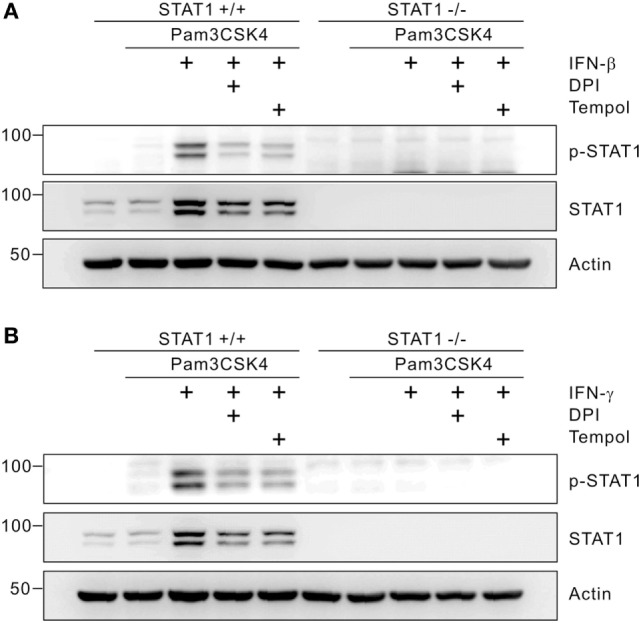
Reactive oxygen species promote signal transducer and activators of transcription 1 (STAT1) phosphorylation. Macrophages from wild-type or STAT1-knockout mice were left untreated or treated with 5 µM of diphenylene iodonium (DPI) or 10 mM of Tempol. After 30 min of pretreatment, cells were incubated with Pam3CSK4 (1 µg/mL) in the absence or presence of interferon (IFN)-β (100 U/mL) **(A)** or IFN-γ (100 ng/mL) **(B)** for 8 h. Cell lysates were then immunoblotted for p-STAT1, STAT1, and actin. Actin was used as a loading control. These results are representative of three independent experiments showing similar results.

Increasing evidence suggests that ROS are involved in intracellular signal transduction affecting multiple signaling pathways including the PG synthesis pathway (19). Recent studies also demonstrated that ROS generation is required for STAT1 activation through phosphorylation in inflamed macrophages (34, 36). Thus, we next investigated whether IFN-mediated ROS generation affects STAT1 activation besides its dependence on STAT1. As shown in Figure 5A, the phosphorylation and level of STAT1 were greatly increased by IFN-β co-treatment, which was diminished by combined treatment with the ROS scavenger Tempol or DPI. Similarly, the elevated phosphorylation and level of STAT1 mediated by IFN-γ co-treatment were prohibited by pretreatment with Tempol or DPI (Figure 5B). Thus, our results suggest that ROS are not only effector molecules that function downstream of STAT1 but also regulate STAT1 levels and activity through a positive-feedback mechanism.

### STAT1 Regulates PGD_2_ and PGE_2_ Production and ROS Generation *In Vivo*

Finally, we evaluated whether STAT1 regulates PG production and ROS generation *in vivo*.

**Figure 6:**
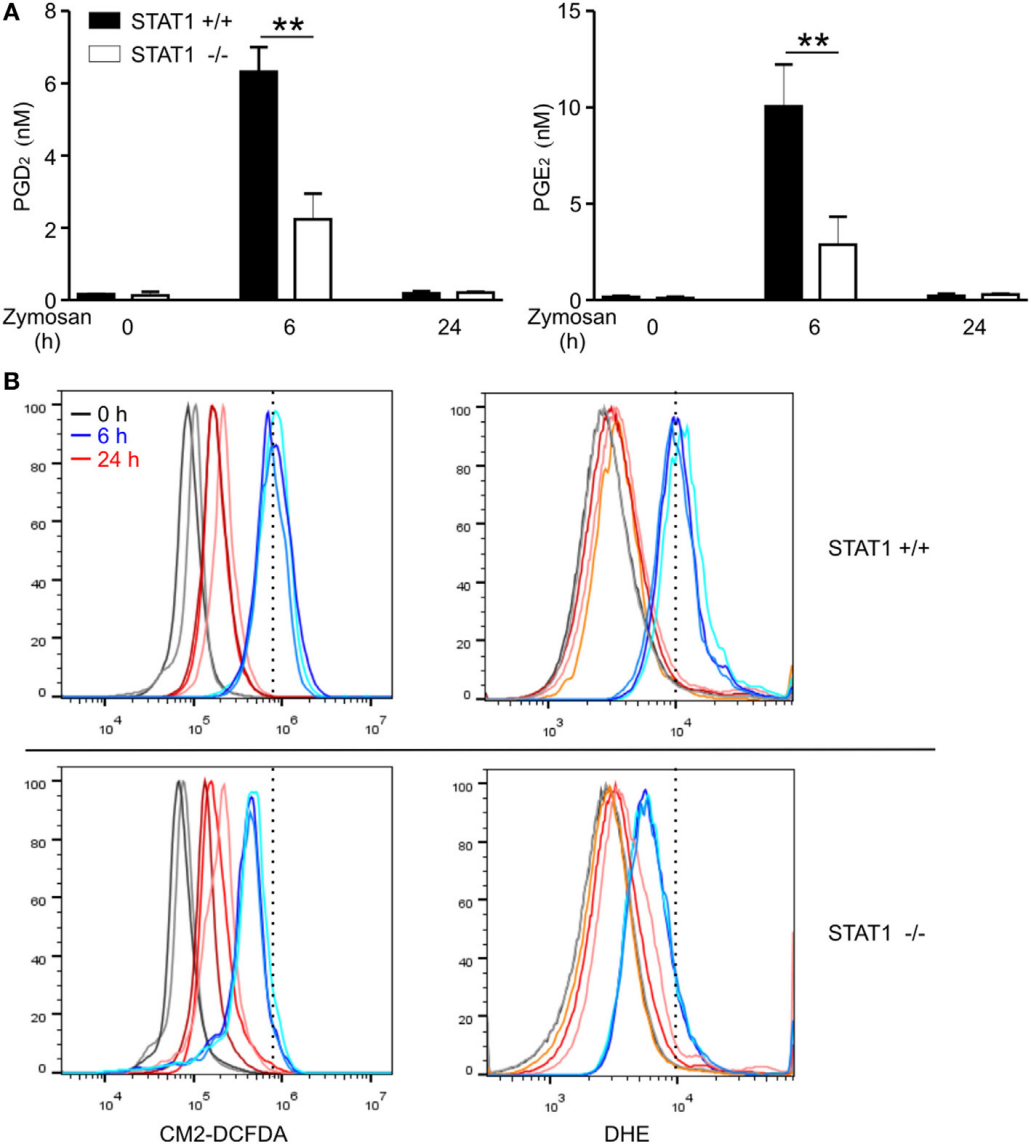
Signal transducer and activators of transcription 1 (STAT1)-dependent PGD_2_ production and reactive oxygen species generation in zymosan-induced peritonitis model. Wild-type or STAT1-knockout mice were left untreated (0 h) or injected i.p. with zymosan (1 mg in 0.5 mL PBS/each mouse) and then sacrificed at the indicated time points. **(A)** Concentrations of PGD_2_ and PGE_2_ in the peritoneal exudates were measured by ELISA. All results are shown as the mean ± SD from three mice in each group. **P < 0.01. **(B)** The peritoneal exudates cells were stained with CM2-DCFDA or DHE for 30 min and the fluorescence intensity was determined by flow cytometry. Each line on the graph represents results from individual mice. These results are representative of two independent experiments.

Zymosan-induced peritonitis is an established model for studying acute and self-resolving inflammation, where pro-inflammatory cytokines including IFNs mediate inflammatory responses (46, 47) and PGD_2_ derived from macrophages contributes to the resolution of peritonitis (13). The production of PGE_2_ from macrophages has been also observed in this model (13). In the absence of zymosan treatment, negligible levels of PGD_2_ and PGE_2_ were detected in the peritoneal lavage (Figure 6A). After i.p. injection of zymosan, the levels of PGD_2_ and PGE_2_ in the peritoneal fluid of wild-type mice were substantially increased at 6 h, with a profound and significant difference between wild-type and STAT1-null mice (Figure 6A). The levels of PGD_2_ and PGE_2_ in the peritoneum returned to basal states at 24 h, in agreement with the self-resolving feature of inflammation in the model. Notably, the levels of PGD_2_ and PGE_2_ production closely correlated with intracellular ROS generation in peritoneal exudate cells, as measured by CM2-DCFDA and DHE (Figure 6B). Overall, these findings suggest that STAT1 is a critical regulator of PG production, including PGD_2_, possibly through an effect on ROS generation under inflammatory conditions.

## Discussion

PGD_2_ has been implicated in diverse inflammatory diseases by serving as a key regulator of inflammation (2). Although the components involved in PGD_2_ biosynthesis have been established, the molecular mechanism underlying PGD_2_ production remains unclear. Previous studies focused on the interaction between PGD_2_ and its receptors (DP1 and DP2) on target cells and understanding the immune-modulating effects of PGD_2_. In this study, we offer a new perspective for the mechanism underlying PGD_2_ production by providing a potential link among IFNs, STAT1, and ROS. Our results revealed that IFNs (IFN-β, IFN-γ) play a priming role but do not activate macrophages for PGD_2_ production on their own, consistent with previous findings (48). Instead, we first demonstrated that IFNs potently enhanced PGD_2_ production from macrophages triggered by TLR stimulation in a STAT1-dependent manner. Using macrophages from STAT1-null mice, we found that potentiation by IFNs relies on STAT1-dependent ROS generation. Interestingly, ROS promoted the activation and protein expression of STAT1, suggesting positive-feedback regulation between STAT1 and ROS for PGD_2_ production. Further, by comparing the results of PGD_2_ with those of PGE_2_, the most well-studied PG, we observed differential regulation of PG synthases (i.e., H-PGDS and mPGES-1) by the IFNs/STAT1 pathway. The relevance of STAT1-dependent regulation of PGD_2_ production *in vivo* was supported by using a model of zymosan-induced peritonitis. Thus, our results suggest that IFN signaling amplifies initial ROS generation by TLR stimulation for PGD_2_ production, with feedback regulation between STAT1 and ROS acting as a central mechanism.

Although IFNs are well-known for their ability to regulate innate and adaptive immune responses under diverse inflammatory conditions (22, 43), their roles in PG production remain largely unknown. A recent study showed that STAT1 was required for PGE_2_ synthesis in human follicular DC-like cells upon IFN-γ stimulation (32). The potentiating effect of IFN-γ on PGD_2_ production from DCs was also reported, but the role of STAT1 in this context was not examined (33). Moreover, the mechanisms of PG production, particularly PGD_2_, mediated by type I IFNs, have not been investigated. In our present study, we uncovered the regulation of PGD_2_ and PGE_2_ production by signaling common to IFN-β and IFN-γ, in which STAT1-dependent ROS generation played a crucial role. It has been shown that stimulation of macrophages with TLR ligands followed by IFNs causes significant increase of STAT1 activation and expression of its target genes including gp91(phox), a key component of the NOX complex, which has a STAT1 binding site in its promoter region (35, 49). In this respect, the dependence of PG production on STAT1 might be accounted for by the requirement for NOX-derived ROS in a STAT1-dependent manner. Supporting this, a NOX inhibitor DPI diminished the production of PGD_2_ and PGE_2_, along with ROS generation, by macrophages upon combined stimulation with Pam3CSK4 and IFNs. We also previously reported that STAT1 signaling modulates intracellular levels of ROS, particularly superoxide that largely comes from NOX, in the context of zVAD/LPS-mediated macrophage death (34). Thus, together with the impaired generation of ROS by STAT1 deficiency, our results suggest that STAT1-dependent ROS are required for IFN-potentiated production of PGD_2_ and PGE_2_.

It is well-known that ROS function as important intracellular signal transducers that contribute to the biosynthesis of PGs from arachidonic acid (19). ROS have been implicated in the inducible expression of mPGES-1 as well as COX-2 and production of PGE_2_ (20, 50, 51). ROS derived from NOX were shown to play an important role in COX-2 expression and subsequent PGE_2_ production during LPS-stimulated microglia (20). LPS-induced ROS generation from NOX2 was also required for the production of PGD_2_ from BMDMs (14). In this study, we found that ROS are required to produce PGD_2_ and PGE_2_ mediated by IFNs, despite the differential regulation of PG synthases; the expression of mPGES-1 is highly inducible, while that of H-PGDS is rather constitutive. Thus, the regulation of H-PGDS expression is not likely the main mechanism of ROS-dependent production of PGD_2_ observed in IFN/Pam3CSK4-treated macrophages. Supporting this notion, ROS was shown to regulate the activity but not expression of H-PGDS in LPS-treated BMDMs, likely serving as a cofactor or contributing to a favorable redox environment for H-PGDS activation (14). In this respect, further studies are required to determine the exact molecular mechanism at which level(s) ROS contribute to the production of PGD_2_.

In addition to the dependence of ROS generation on STAT1, ROS have been shown to regulate the activation of STAT1 *via* phosphorylation (36, 37, 52). ROS, by inhibiting phosphatases, can activate JAK2 and tyrosine kinase 2 kinases, leading to activation of STAT (52). Treatment with IFN-γ results in a significant increase in ROS generation and STAT1 phosphorylation, which is abrogated by pretreatment with ROS scavenger (37). TLR3-induced ROS generation from NOX is required for the activation of NF-κB and STAT1-mediated immune responses in macrophages (36). Corroborating these findings, we found that ROS generated by the IFN/STAT1 pathway, in turn, promoted STAT1 phosphorylation and expression (Figure 5). Thus, our results suggest the cross-regulation but not hierarchical regulation between STAT1 and ROS in mediating the production of PGD_2_ and PGE_2_ in inflamed macrophages. This cross-regulation between STAT1 and ROS was also observed in our previous study of zVAD/LPS-mediated macrophage death showing that STAT1 signaling amplifies intracellular oxidative stress through a positive-feedback mechanism, which is interrupted by activated caspases (34).

In the present study, we found that both IFN-β and IFN-γ substantially potentiated the production of PGD_2_ and PGE_2_ by Pam3CSK4-treated macrophages in a STAT1-dependent manner. It has been shown that the potentiating effect by IFNs is context-dependent, depending on the IFN, TLR ligand, and cytokine produced. IFN-γ potently enhances macrophage production of TNF-α, IL-6, and IL-12 but suppresses that of IL-10 in response to various TLR ligands including Pam3CSK4 (42, 53). IFN-γ also cooperates with Pam3CSK4 in vascular endothelia cells for enhanced expression of iNOS and nitric oxide production *via* STAT1 activation (54). In comparison, IFN-β potentiates macrophage production of TNF-α, IL-6, and IL-10 but negatively regulates that of IL-12 (42) in a manner dependent on IL-10 production (55, 56). IFN-β in combination with LPS cannot enhance TNF-α production (42, 53). Thus, the potentiation of PG production by IFNs in response to Pam3CSK4, LPS, and zymosan in our results (Figure 1 and data not shown) and others (33) suggests that this is a conserved feature of stimulation through diverse IFNs and TLR ligands, warranting further investigation.

In summary, we demonstrated the potentiating effect of IFNs, particularly type I IFN (IFN-β), on the production of PGD_2_ and PGE_2_ under inflammatory conditions. This potentiation by IFNs was attributed to cross-regulation between STAT1 and ROS in a positive-feedback manner. The importance of STAT1-dependent regulation of PGs was supported by using a model of zymosan-induced peritonitis in this study and likely a model of bronchial asthma in a previous study (57). In the previous study, inhibition of STAT1-attenuated allergen-induced airway inflammation, particularly eosinophilic inflammation, which is closely associated with PGD_2_ dysregulation. Thus, STAT1-dependent PG regulation under inflammatory conditions may be relevant in various settings of inflammatory disorders, the impact, and therapeutic potential of which require further investigation.

## Ethics Statement

All animal experiments in this study were approved by Institutional Animal Care and Use Committee (IACUC) of Asan Institute for Life Science with license number of 2012-02-074 and done in accordance with the approved guidelines set forth by IACUC.

## Author Contributions

J-YK, G-EC, and HK conceived, designed, and performed the experiments and analyzed the data. HY provided the analytical tools. HS provided overall coordination and supervision of the study. HK and J-YK wrote the manuscript.

## Conflict of Interest Statement

The authors declare that the research was conducted in the absence of any commercial or financial relationships that could be construed as a potential conflict of interest.
