# Supplementary material for: Interferon Potentiates Toll-Like Receptor-Induced Prostaglandin D2 Production through Positive Feedback Regulation between Signal Transducer and Activators of Transcription 1 and Reactive Oxygen Species
Source: Front Immunol. 2017 Dec 4;8:1720. doi: 10.3389/fimmu.2017.01720 (PMC5723016; doi:10.3389/fimmu.2017.01720)
Supplement: Supplementary file 1 [file Presentation_1.PDF]

Supplementary Material

Interferon Potentiates TLR-Induced Prostaglandin D_2 Production through Positive Feedback Regulation between STAT1 and Reactive Oxygen Species

Running title: IFN/STAT1/ROS Pathway Mediates PGD_2 Production

Ji-Yun Kim[1,#], Go-Eun Choi[1,2,#], Hyun Ju Yoo[3] and Hun Sik Kim[1,4,5,*]

[1]Department of Biomedical Sciences, Asan Medical Center, University of Ulsan College of Medicine, Seoul, Korea; [2]Instituteof Convergence Bio-Health, Dong-A University, Busan, Korea; [3]Biomedical Research Center, Asan Institute of Life Sciences, Department of Convergence Medicine, Asan Medical Center, University of Ulsan College of Medicine, Seoul, Korea; [4]Department of Microbiology, Asan Medical Center, University of Ulsan College of Medicine, Seoul, Korea; [5]Cellular Dysfunction Research Center, Asan Medical Center, University of Ulsan College of Medicine, Seoul, Korea

[#]These authors contributed equally to this work

***Correspondence:**

Hun Sik Kim

E-mail: hunkim@amc.seoul.kr

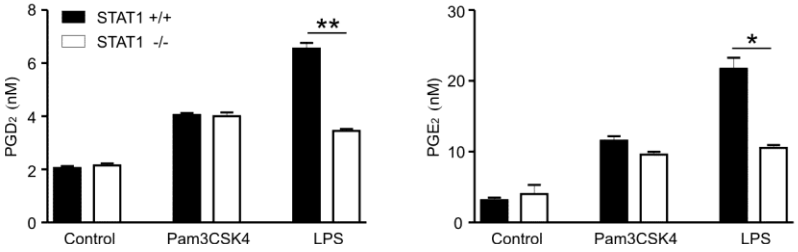

Supplementary Figure 1. Effect of Pam3CSK4 or LPS on PG production by STAT1 deficiency.

Peritoneal macrophages were left untreated (control) or treated with Pam3CSK4 (1 μg/mL) or LPS (100 ng/mL) for 24 h. The production of PGD_2 and PGE_2 in the culture supernatant was determined by ELISA. Data represent the means ± SD of three independent experiments. *P<0.05, **P<0.01.

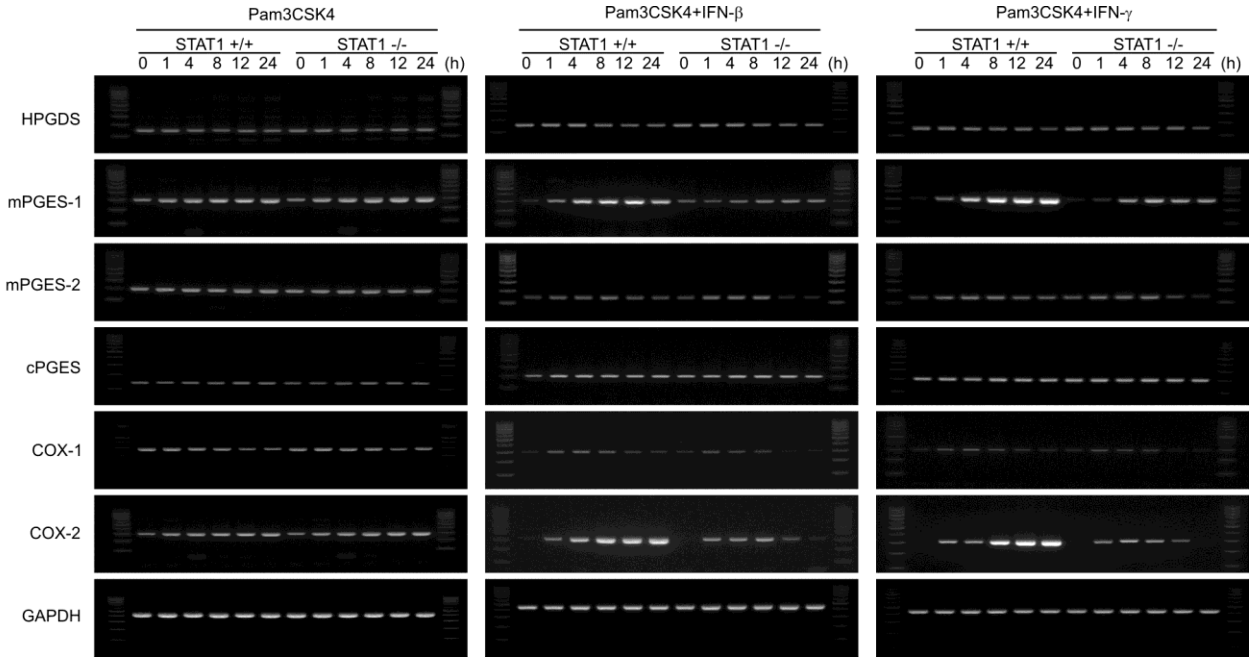

Supplementary Figure 2. Effect of STAT1 on mRNA expression of PGDS, PGES and COX isoforms.

Macrophages were incubated with 1 μg/mL of Pam3CSK4 for the indicated times in the absence or presence of 100 U/mL of IFN-β or 100 ng/mL of IFN-γ. The mRNA levels of H-PGDS, mPGES-1, mPGES-2, cPGES, COX-1, COX-2, and GAPDH were analyzed by RT-PCR. Shown are representative results of at least three independent experiments showing similar results.

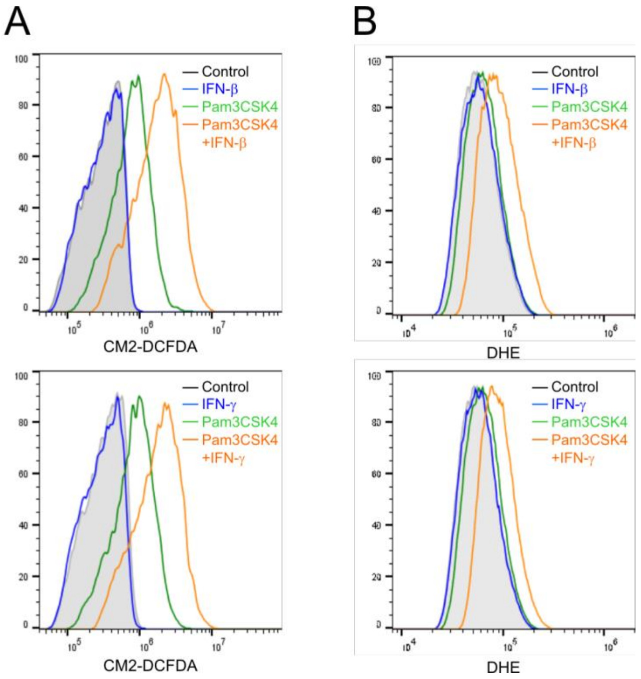

Supplementary Figure 3. Priming role of IFNs in ROS generation.

Peritoneal macrophages were left untreated (control) or treated with IFN-β (100 U/mL) or IFN-γ (100 ng/mL), either individually or in combination with Pam3CSK4 (1 μg/mL) for 6 h. Cells were then stained for 30 min with CM2-DCFDA (5 μM) (A) or DHE (20 μM) (B). ROS generation was analyzed by flow cytometry. Data are representative of three independent experiments.
